# Computational identification of novel SIRT4 inhibitors for diabetic nephropathy using pharmacophore modeling, molecular simulations, and DFT calculations

**DOI:** 10.1371/journal.pone.0336948

**Published:** 2025-11-17

**Authors:** Wenxiang He, Jianwu Chen

**Affiliations:** 1 Department of General Medicine, The Third People’s Hospital of Hubei Province, Wuhan, Hubei province, China,; 2 Department of Nephrology, The Third People's Hospital of Hubei Province, Wuhan, Hubei province, China; Universiti Teknologi Malaysia, MALAYSIA

## Abstract

Sirtuin 4 (SIRT4) plays a critical role in regulating oxidative stress, apoptosis, and mitochondrial dysfunction in diabetic nephropathy (DN). This study employed a multi-step in silico strategy to identify novel SIRT4 modulators with potential therapeutic relevance for DN. A ligand-based pharmacophore model was developed using UBCS182, followed by virtual screening of 3,285 compounds from major chemical libraries. Molecular docking revealed strong binding affinities (−9.46 to −8.41 kcal/mol), with CSC057320968, PubChem-162316407, and ChemDiv-V013-1548 emerging as top candidates. ADMET analysis confirmed their favorable pharmacokinetic and toxicity profiles. Subsequent 200 ns molecular dynamics simulations demonstrated the stability of protein–ligand complexes, with CSC057320968 exhibiting the most stable interaction profile based on RMSD, RMSF, Rg, and contact frequency analyses. Principal component analysis and free energy landscapes indicated conformational rigidity and energetic favorability for CSC057320968. Density Functional Theory (DFT) analysis further validated its reactivity and chemical softness, supporting its potential as a lead scaffold. This integrated computational pipeline provides novel insights into SIRT4 modulation and offers a rational framework for targeting mitochondrial dysfunction in DN.

## 1. Introduction

Diabetic nephropathy (DN) is a leading cause of end-stage renal disease worldwide, driven by persistent hyperglycemia-induced damage to renal tissues. Among the critical cellular components affected, podocytes, specialized epithelial cells of the glomerular filtration barrier are particularly vulnerable to oxidative stress, mitochondrial dysfunction, and inflammation [1–3]. These pathological events contribute significantly to the progression of DN and the eventual decline in renal function [4,5]. Targeting mitochondrial regulators that preserve podocyte integrity represents a promising therapeutic strategy in DN, yet remains underexplored.

Recent studies have spotlighted Sirtuin 4 (SIRT4), a mitochondrial ADP-ribosyltransferase, for its pivotal role in cellular metabolism, redox balance, and apoptotic regulation. SIRT4 suppresses reactive oxygen species (ROS), maintains mitochondrial membrane potential, and modulates key apoptotic markers such as NOX1, Bax, and Bcl-2 [6–8]. It also inhibits glutamate dehydrogenase (GDH), thereby protecting against ATP overproduction and preserving mitochondrial efficiency under hyperglycemic stress [9–11]. Additionally, SIRT4 has been shown to downregulate pro-inflammatory cytokines like TNF-α, IL-1β, and IL-6, indicating its dual role in mitochondrial regulation and inflammatory suppression in diabetic kidneys [12,13].

Despite these protective functions, the pharmacological targeting of SIRT4 remains in its infancy. Most functional studies have relied on gene editing or overexpression models, with limited exploration into structure-based small-molecule drug discovery. To date, no selective SIRT4 modulators have progressed to clinical evaluation, and computational approaches to identify SIRT4 inhibitors are notably scarce [14,15].

It is important to note that existing literature often reports SIRT4 overexpression as protective against DN progression. However, the broader therapeutic concept is that SIRT4 modulation, rather than simple activation, may hold therapeutic value depending on disease stage, tissue type, and metabolic context. Inhibitors such as UBCS182 have provided structural templates for computational studies and may uncover chemical scaffolds capable of fine-tuning SIRT4 activity. Therefore, in this study, we used UBCS182 as a reference to develop a pharmacophore model and identify novel small-molecule modulators classified here as inhibitors through a comprehensive in silico strategy. We hypothesized that a multi-tiered virtual screening approach could uncover potent and selective SIRT4 modulators with promising pharmacokinetic and electronic profiles.

Our methodology combined: pharmacophore modeling based on the known SIRT4 inhibitor UBCS182, virtual screening across major chemical databases, molecular docking and ADMET profiling to identify drug-like candidates, 200 ns molecular dynamics (MD) simulations to validate binding stability, and Density Functional Theory (DFT) calculations to assess electronic properties and reactivity. This study introduces structurally novel scaffolds that demonstrate strong interactions with SIRT4 and provides a replicable computational pipeline for discovering mitochondria-targeted therapeutics in DN.

## 2. Methods

### 2.1 Pharmacophore modelling and virtual screening

To identify novel small-molecule modulators of SIRT4, a ligand-based pharmacophore model was developed using the known inhibitor UBCS182 [16]. The chemical interactions between UBCS182 and the active site residues of SIRT4 were analyzed to extract critical pharmacophoric features, including aromatic rings, hydrophobic groups, and hydrogen bond acceptors. This pharmacophore model was constructed using the Pharmit server, which allows for interactive pharmacophore modeling and virtual screening. The model served as a query to screen seven major compound libraries containing PubChem, ZINC, ChEMBL, ChemDiv, MolPort, LabNetwork, and Enamine comprising millions of commercially available drug-like molecules. To ensure high drug-likeness and bioavailability, screening filters were applied in accordance with Lipinski’s Rule of Five: molecular weight less than 500 Da, LogP less than 5, hydrogen bond acceptors fewer than 10, hydrogen bond donors fewer than 5, and topological polar surface area (TPSA) less than 140 Å². A total of 3,285 hits were obtained and subjected to structural refinement and energy minimization using LigPrep (Schrödinger Suite), which generated relevant tautomers, stereoisomers, and ionization states for molecular docking [17].

### 2.2 Molecular docking

The crystal structure of human SIRT4 (PDB ID: 5OJ7) was retrieved from the Protein Data Bank. This structure was selected because it represents the highest-quality experimentally resolved SIRT4 model available, determined by X-ray diffraction at 1.58 Å resolution with excellent refinement statistics (R-work = 0.152; R-free = 0.186). Importantly, 5OJ7 contains the co-crystallized ligand ADP-ribose, which precisely defines the active site environment. The structure was processed using the Protein Preparation Wizard in Maestro [18]. The preprocessing steps included assignment of correct bond orders, addition of hydrogens, generation of zero-order bonds to metal centers, and removal of crystallographic water molecules beyond 5 Å from the binding site. The protonation states of ionizable residues were optimized using Epik at physiological pH (7.0), and any missing loops or side chains were reconstructed using Prime [19]. The entire structure was minimized using the OPLS3e force field [20]. A receptor grid was generated around the SIRT4 active site using centroid coordinates (X = 15.37, Y = 11.43, Z = 41.25 Å). The grid box was defined with dimensions of 20 × 20 × 20 Å to fully encompass the binding pocket and adjacent flexible regions. Ligands were docked using Glide in Standard Precision (SP) mode, generating up to 10 poses per ligand, with the best-ranked pose retained for further analysis. GlideScore was employed as the scoring function, which integrates electrostatic, van der Waals, hydrophobic, hydrogen-bonding, and desolvation terms [21]. The docking results were analyzed, and drugs were selected based on glide scores.

### 2.3 Post docking analysis

The screened hits were docked to the SIRT4 receptor to obtain the binding affinities. The top ten hits were selected based on glide scores and their molecular interactions were analyzed by using Discovery Studio [22].

### 2.4 Druglikeness and toxicity analysis

Drug erosion is attributed to toxicity and poor pharmacokinetics issues [23]. To assess the pharmacokinetic viability and safety of the docked hits, in silico ADMET profiling was performed using the OSIRIS Property Explorer [24]. Molecular descriptors such as molecular weight, LogP, TPSA, solubility (LogS), and drug-likeness scores were computed. The QikProp tool was used to calculate the QPlogHERG and QPlogBB descriptors. Additionally, compounds were screened for toxicity risks including mutagenicity, tumorigenicity, irritancy, and reproductive toxicity. Candidates that satisfied all drug-likeness filters and exhibited low toxicity risk were shortlisted for dynamic simulations.

### 2.5 MD simulation

The structural stability and binding persistence of selected protein-ligand complexes were evaluated using 200 ns molecular dynamics (MD) simulations conducted in Desmond [25]. The systems were solvated in an orthorhombic periodic box using the TIP3P water model, and counterions (Na⁺ and Cl⁻) were added to neutralize the system [26]. A salt concentration of 0.15 M NaCl was used to mimic physiological ionic strength. The systems were energy minimized and equilibrated using a predefined relaxation protocol. Simulations were carried out in the NPT ensemble at 300 K temperature and 1 atm pressure, using the Nose–Hoover thermostat and Martyna–Tobias–Klein barostat to maintain thermodynamic stability [27]. Trajectories were recorded every 50 ps, and key metrics including root mean square deviation (RMSD), root mean square fluctuation (RMSF), and hydrogen bond occupancy were analyzed using the Simulation Interaction Diagram tool to evaluate system behavior and ligand stability within the binding site.

### 2.6 Density functional theory (DFT) calculations

The selected hit compounds were subjected to quantum chemical analysis to evaluate their frontier molecular orbitals (HOMO and LUMO) and global reactivity descriptors. Geometry optimization of each compound was performed using the Density Functional Theory (DFT) method with the B3LYP functional and the 6-311G(d,p) basis set, as implemented in the Gaussian 16 software package [28]. The calculations were carried out in a water environment using the solvent model, ensuring a more accurate representation of the compounds’ behavior in physiological conditions. Default convergence criteria were applied without imposing any symmetry constraints.

## 3. Results

### 3.1 Virtual screening

**Fig 1:**
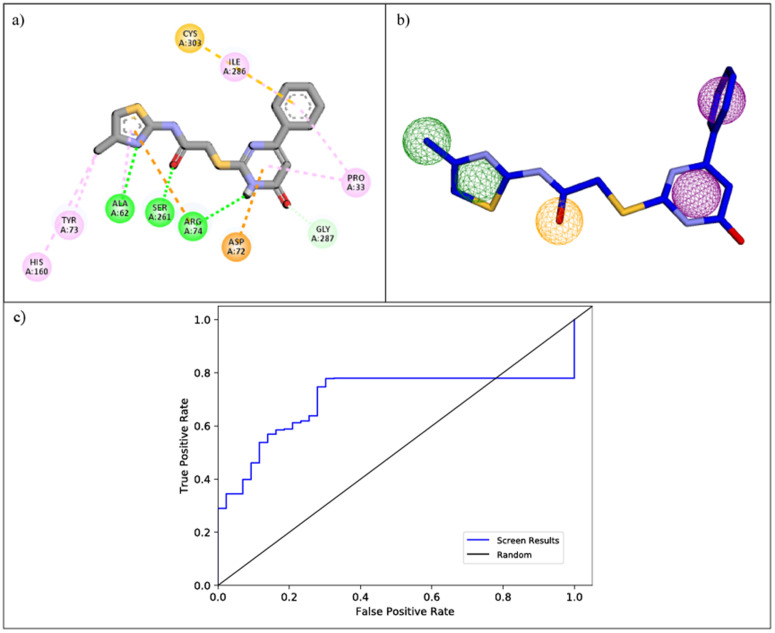
Pharmacophore development based on UBCS182 binding to SIRT4. (a) Molecular interactions of UBCS182 with the SIRT4 active site, showing hydrogen bonds (green dashed lines), hydrophobic contacts (orange dashed lines), and π–π interactions (purple dashed lines) with key residues. (b) Structure-based pharmacophore model generated using Pharmit. Aromatic rings (purple spheres), hydrophobic groups (green spheres), and a hydrogen bond acceptor (orange sphere). (c) Receiver operating characteristic (ROC) curve for pharmacophore validation against active and decoy sets.

To initiate the identification of novel modulators for SIRT4, the molecular interactions of the reference inhibitor UBCS182 with the SIRT4 active site were analyzed in detail. As shown in Fig 1a, UBCS182 was found to establish multiple stabilizing interactions within the binding pocket, including key hydrogen bonds with residues such as Asp72, Arg74, and Ser261, π–π stacking with Tyr73, and hydrophobic contacts with Ala62, Pro33, and Cys303. These observations highlighted the molecular features crucial for effective SIRT4 binding and guided the design of a pharmacophore model. Using the Pharmit web server, a five-point pharmacophore hypothesis was generated based on the binding conformation of UBCS182. The model incorporated two aromatic rings (depicted as purple spheres), two hydrophobic groups (green spheres), and one hydrogen bond acceptor (orange sphere), capturing the essential interaction features required for affinity toward the SIRT4 binding pocket (Fig 1b). To evaluate the predictive performance of the developed pharmacophore, validation was conducted using a dataset comprising known SIRT4 actives and property-matched decoys. The receiver operating characteristic (ROC) curve yielded an area under the curve (AUC) of 0.70, indicating moderate ability to distinguish actives from inactives (Fig 1c). Early recognition ability was reflected in the enrichment factor at 1% (EF1% = 1.03), while the Boltzmann-enhanced discrimination of receiver operating characteristic metric (BEDROC160.9 = 1.00) further confirmed the model’s sensitivity to early active retrieval. The area under accumulation curve (AUAC = 0.51) supported balanced active recovery across the ranked dataset. Collectively, these validation metrics demonstrate that the pharmacophore model is both reliable and capable of enriching true actives during virtual screening. The spatial coordinates and radii of these features are listed in Table 1, and they define a 3D arrangement that mirrors the binding conformation of UBCS182 in the crystal structure. This pharmacophore model was subsequently used to screen seven large chemical libraries integrated into the Pharmit platform: PubChem, ZINC, ChEMBL, ChemDiv, Molport, LabNetwork, and Enamine. To ensure drug-likeness and alignment with pharmacokinetic guidelines, the following filters were applied during screening: molecular weight < 500 Da, LogP < 5, number of hydrogen bond donors (HBD) < 5, hydrogen bond acceptors (HBA) < 10, and topological polar surface area (TPSA) < 140 Å². These thresholds are consistent with Lipinski’s Rule of Five and enhance the likelihood of identifying orally bioavailable candidates. The virtual screening process yielded a total of 3,285 unique hit compounds, with the highest contributions coming from the PubChem (n = 1,423), ZINC (n = 751), and Molport (n = 372) databases, as summarized in Table 2. These filtered hits were subsequently exported and subjected to 3D structure standardization and optimization using LigPrep. The resulting library was then prepared for structure-based docking against the SIRT4 receptor.

**Table 1:** Three-dimensional coordinates of the pharmacophore features derived from UBCS182 interaction with SIRT4.

Features	X	Y	Z	Radius
Hydrophobic	17.40	16.17	41.59	1 Å
Hydrophobic	19.97	16.92	42.03	1 Å
Aromatic	14.28	6.14	39.48	1 Å
Aromatic	12.01	8.88	41.76	1 Å
Hydrogen Acceptor	14.08	14.30	41.77	1 Å

**Table 2:** Distribution of virtual screening hits across seven chemical databases.

Sr.	Databases	Hits
1	CHEMBL	117
2	ChemDiv	183
3	Molport	372
4	PubChem	1423
5	LabNetwork	180
6	ZINC	751
7	Enamine	259
	Total	3285

### 3.2 Molecular Docking and Interaction Analysis

**Fig 2:**
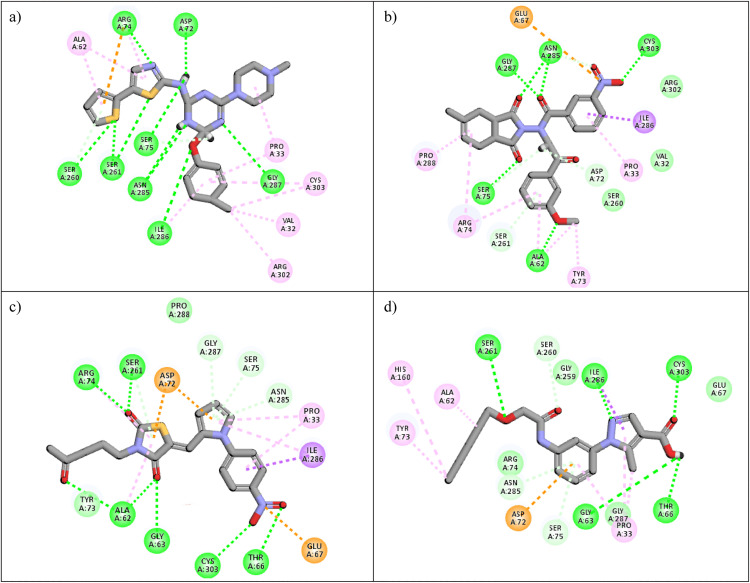
2D interaction diagrams of top four ligands docked to SIRT4 active site. (a) PubChem-152882304; (b) PubChem-125701238; (c) PubChem-142748620; (d) CSC057320968. Hydrogen bonds (green), π-interactions (pink), hydrophobic contacts (orange), and van der Waals forces are shown. Labels indicate key interacting residues across the active site pocket.

Following the virtual screening of 3,285 compounds, molecular docking was performed to evaluate the binding affinities of each hit against the active site of the SIRT4 protein [29]. The docking process, executed using Glide SP, yielded binding scores ranging from –9.46 to –8.41 kcal/mol, indicating generally strong affinities for the selected compounds. The top ten ligands with the most favorable docking scores are summarized in Table 3. Among them, PubChem-152882304 emerged as the most promising candidate, achieving a docking score of –9.466 kcal/mol, followed closely by PubChem-125701238 (–9.315 kcal/mol) and PubChem-142748620 (–8.814 kcal/mol). These scores suggest a robust interaction with key residues in the SIRT4 active site, justifying their selection for further analysis. To further characterize the binding profiles of these ligands, detailed interaction analysis was conducted using Discovery Studio. As illustrated in Fig 2, the top compounds engaged a diverse set of non-covalent interactions, including hydrogen bonds, π–π stacking, π-sigma interactions, and alkyl or hydrophobic contacts, all of which contribute to complex stability and binding specificity [30]. PubChem-152882304 formed an extensive hydrogen-bonding network involving residues Arg74, Asp72, Ser260, Ser261, Ser75, Asn285, Ile286, and Gly287. It also established five alkyl interactions with Ala62, Pro33, Cys303, Val32, and Arg302 (Fig 2a). This interaction profile underscores a high degree of spatial complementarity within the binding pocket. PubChem-125701238, ranked second by docking score, formed seven hydrogen bonds with Gly287, Asn285, Cys303, Ser75, Ala62, Ser261, and Asp72. Additionally, it engaged in a π-sigma interaction with Ile286 and established multiple van der Waals and alkyl contacts with Pro33, Tyr73, Arg74, and Pro288 (Fig 2b), indicating both depth and breadth of interaction. PubChem-142748620 exhibited nine hydrogen bonds, notably with Arg74, Ser261, Ala62, Gly73, Cys303, Thr66, Asn285, Ser75, and Gly287. It also displayed a π-sigma interaction with Ile286 and one alkyl contact with Pro33 (Fig 2c). The multiplicity of interactions suggests strong binding stability despite its relatively lower docking score compared to the top two ligands. CSC057320968, another high-ranking compound, formed eight hydrogen bonds with Ser261, Ser260, Ile286, Cys303, Thr66, Gly63, Ser75, and Asn285. It also established four alkyl interactions involving Ala62, His160, Tyr73, and Pro33, and several van der Waals contacts (Fig 2d). This compound’s interaction profile was particularly notable for its engagement with residues across the binding pocket, suggesting enhanced structural anchoring. The interaction data for the remaining compounds including ZINC000408642164, PubChem-138579602, PubChem-162316407, PubChem-165547127, ChemDiv-V013-1548, and PubChem-92742026 are summarized in Table 4. These ligands displayed consistent interactions with conserved residues such as Ser75, Ser261, Asn285, and Cys303, further validating the pharmacophore-guided screening approach. Overall, the top ten ligands demonstrated favorable docking profiles and strong interaction patterns with critical residues within the SIRT4 active site. The combination of electrostatic, hydrophobic, and hydrogen-bonding interactions suggests these compounds possess the structural requirements for stable and selective binding to SIRT4.

**Table 3:** Docking scores of the top 10 ligands identified from virtual screening against the SIRT4 active site.

No.	Compound Code	Structures	Docking scores (kcal/mol)
1	PubChem-152882304	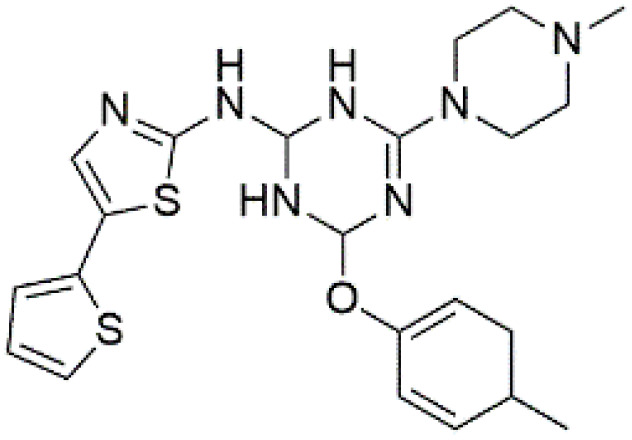	−9.46
2	PubChem-125701238	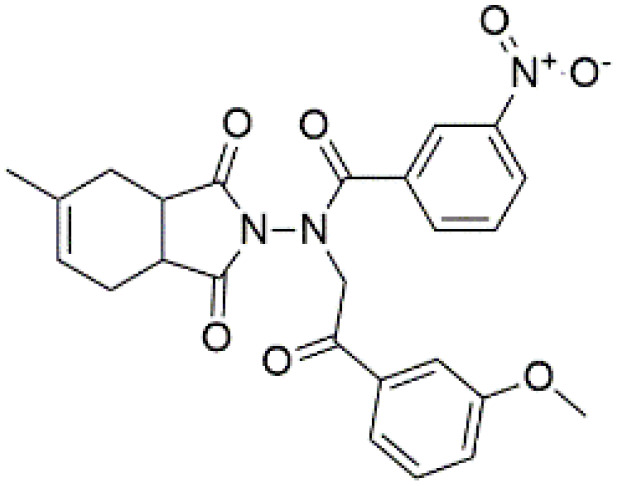	−9.31
3	PubChem-142748620	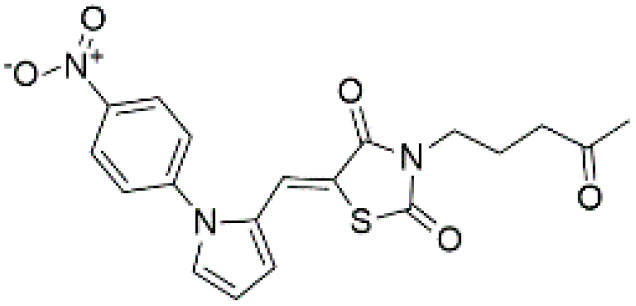	−8.81
4	CSC057320968	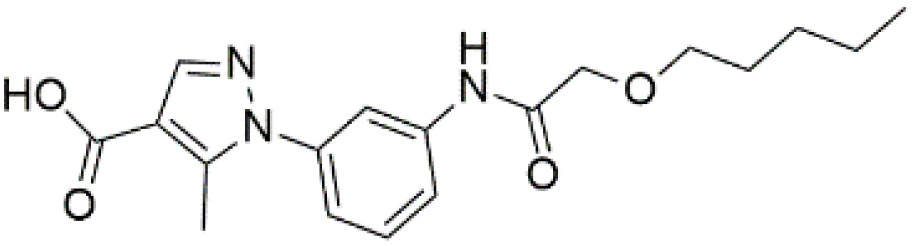 >	−8.68
5	ZINC000408642164	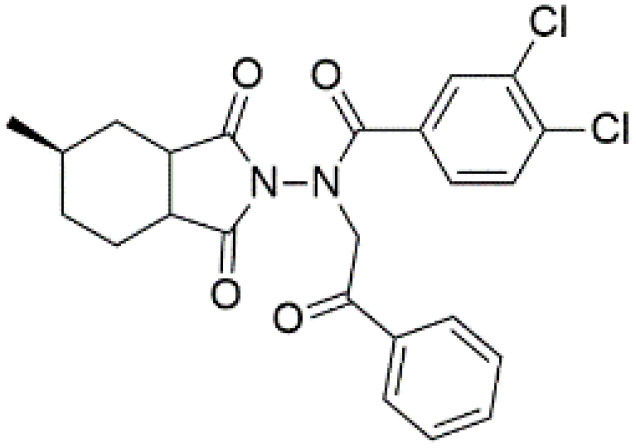	−8.61
6	PubChem-138579602	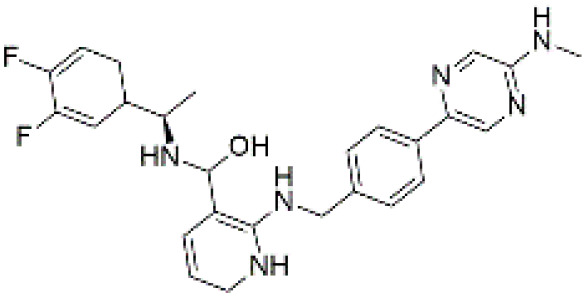	−8.60
7	PubChem-162316407	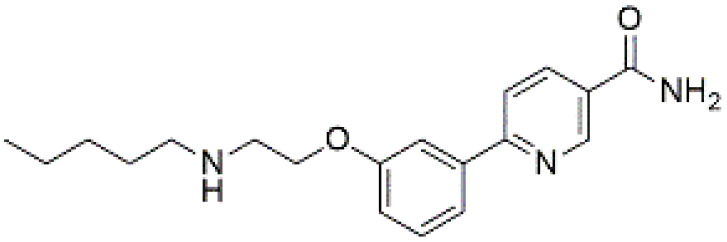	−8.56
8	PubChem-165547127	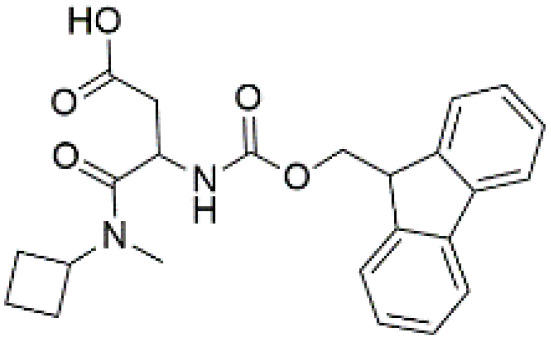	−8.56
9	ChemDiv-V013-1548	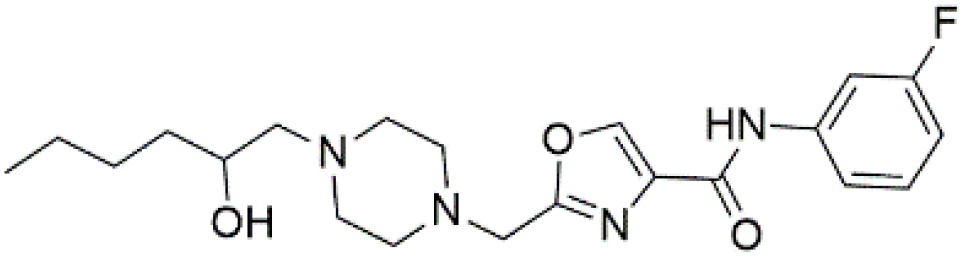	−8.45
10	PubChem- 92742026	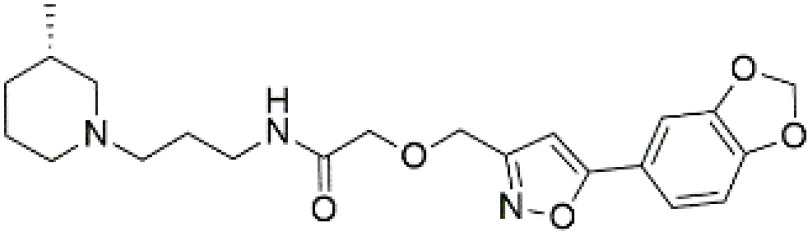	−8.41

**Table 4:** Summary of key molecular interactions between selected ligands and SIRT4 active site residues.

Sr.	Compound code	Molecular Interactions
1	ZINC000408642164	**Hydrogen Bond:** Asn285, Ser75, Gly287, Asp72, Ser261 **Pi-Sigma:** Ile286 **Van der Waal:** Tyr73, Ser260, Gly63, Thr66 **Alkyl:** Arg74, Ala62, Val32, Pro33, Pro288
2	PubChem-138579602	**Hydrogen Bond:** Arg74, Ser260, Ser261, Gln142, Ser75 **Van der Waal:** Pro288, Ile286, Pro33, Gly287, Asn285 **Alkyl:** Tyr73, Ala62, His160
3	PubChem-162316407	**Van der Waal:** Ser261, Ser260, **Hydrogen Bond:** Ser75, Ile286, Glu67, Thr66, Cys303, Asn285, Arg74 **Alkyl:** Pro36, Pro33
4	PubChem-165547127	**Van der Waal**: Glu67, Pro33, Thr66 **Hydrogen Bond:** Asn285, Ser75, Asp72, Ser261, Arg74, Gly63, Ile286 **Alkyl:** Cys303, Pro288
5	ChemDiv-V013-1548	**Hydrogen Bond:** Gly61, Gly259, Ile286, Ser260, Ser261, Arg74, Gln142, Tyr73 **Alkyl:** Phe232, Val144, Val230, Ala62
6	PubChem- 92742026	**Hydrogen Bond:** Gly63, Glu67, Cys303, Gly287, Asn285, Ser75, Asp72, Ser261, Thr66 **Pi-Sigma:** Ile286 **Alkyl:** Val264, Tyr81, Tyr73, Ala62, Val32, Arg302, Pro33

### 3.4 ADMET analysis

To assess the pharmacokinetic suitability and safety of the top-ranked compounds, an in silico ADMET (Absorption, Distribution, Metabolism, Excretion, and Toxicity) profiling was conducted using OSIRIS Property Explorer and QikProp descriptors. Key pharmacokinetic parameters evaluated included molecular weight (MW), partition coefficient (LogP), topological polar surface area (TPSA), and aqueous solubility (LogS), which together inform oral bioavailability [31]. Compounds with TPSA values below 160 Å² are generally considered favorable for intestinal absorption [32]. Among the selected hits, the majority complied with standard drug-likeness filters, including MW < 500 Da, LogP < 5, TPSA < 160 Å², and LogS > –5, suggesting good intestinal permeability and solubility profiles. Notably, PubChem-152882304 exceeded the LogP threshold (5.55), indicating potential absorption issues due to excessive lipophilicity. Similarly, PubChem-142748620 presented a LogS of –6.35, signifying poor water solubility, which may limit its bioavailability despite acceptable MW and TPSA values. The TPSA values for all compounds ranged between 74.76 and 133.5 Å², well below the 160 Å² threshold typically associated with good oral bioavailability. CSC057320968, PubChem-162316407, and ChemDiv-V013-1548 displayed particularly favorable profiles with balanced LogP, moderate MW, and high solubility highlighting their potential as orally bioavailable drug candidates. In addition to physicochemical attributes, the compounds were evaluated for drug-likeness and drug score. The drug-likeness metric reflects structural similarity to known drugs, whereas the drug score integrates several parameters including solubility, hydrophilicity, MW, and toxicity risk. CSC057320968 and ChemDiv-V013-1548 showed the highest drug scores (0.44 and 0.68, respectively), suggesting a favorable balance of pharmacokinetic traits and safety profile. The higher the drug score for a compound, the higher the potential of that compound to be developed into a medication [33]. To further evaluate safety profiles, hERG inhibition (QPlogHERG) and blood–brain barrier (BBB) permeability (QPlogBB) predictions were incorporated. QPlogHERG values below –5 is generally associated with increased risk of cardiotoxicity due to hERG potassium channel inhibition. Among the compounds, PubChem-152882304 (–8.39) and PubChem-138579602 (–8.03) showed significant predicted liability, while CSC057320968 (–4.02) and ChemDiv-V013-1548 (–6.08) fell within acceptable or borderline ranges. Regarding BBB permeability, QPlogBB values between –1.0 and 0.3 typically indicate moderate penetration. CSC057320968 (–1.68) and PubChem-162316407 (–1.18) were predicted as weakly permeable, reducing potential CNS-related side effects and favoring peripheral selectivity, which is desirable for diabetic nephropathy therapy (Table 5). Toxicity predictions revealed a few compounds with potential risks. PubChem-152882304, PubChem-165547127, and PubChem-92742026 were flagged for high or medium mutagenicity, while PubChem-152882304 and PubChem-125701238 were associated with high reproductive toxicity. PubChem-142748620 was identified with a high irritant risk. These toxicity flags may present obstacles for further development, especially for compounds with borderline pharmacokinetic properties (Table 6). Based on the combined ADMET and toxicity profiles, three compounds CSC057320968, PubChem-162316407, and ChemDiv-V013-1548 were selected for molecular dynamics (MD) simulations, alongside the reference compound UBCS182. These ligands were prioritized for their optimal balance of binding affinity, physicochemical characteristics, and low predicted toxicity risk.

**Table 5:** Predicted physicochemical and ADME properties of top-ranked SIRT4 ligands.

Compound codes	MW	LogP	TPSA	LogS	Druglikeness	Drug score	QPlogHERG	QPlogBB
PubChem-152882304	471	5.55	133.5	−4.11	9.45	0.17	−8.39	0.43
PubChem-125701238	477	0.7	129.8	−4.43	−1.64	0.22	−5.96	−1.90
PubChem-142748620	399	1.78	130.5	−6.35	−8.63	0.16	−6.13	−2.16
CSC057320968	345	1.69	93.45	−2.8	6.15	0.44	−4.02	−1.68
ZINC000408642164	472	3.0	74.76	−4.69	1.78	0.37	−5.95	−0.44
PubChem-138579602	480	2.23	94.13	−4.2	2.98	0.45	−8.03	−0.46
PubChem-162316407	327	2.63	77.24	−3.6	4.01	0.41	−6.97	−1.18
PubChem-165547127	422	2.37	95.94	−4.91	−3.2	0.11	−3.31	−1.50
ChemDiv-V013-1548	404	2.33	81.84	−2.26	0.58	0.68	−6.08	−1.06
PubChem- 92742026	415	2.46	86.06	−3.81	3.58	0.28	−5.28	−0.69

**Table 6:** Predicted toxicity risk assessment of top-ranked SIRT4 ligands.

Compound codes	Mutagenic	Tumorigenic	Irritant	Reproductive effect
PubChem-152882304	High	Passed	Passed	High
PubChem-125701238	Passed	Passed	Passed	High
PubChem-142748620	Passed	Passed	High	Passed
CSC057320968	Passed	Passed	Passed	Passed
ZINC000408642164	Passed	Passed	Passed	Passed
PubChem-138579602	Passed	Passed	Passed	Passed
PubChem-162316407	Passed	Passed	Passed	Passed
PubChem-165547127	High	High	Passed	Passed
ChemDiv-V013-1548	Passed	Passed	Passed	Passed
PubChem- 92742026	High	Medium	Medium	passed

### 3.6. MD simulation studies

#### 3.6.1. RMSD.

**Fig 3:**
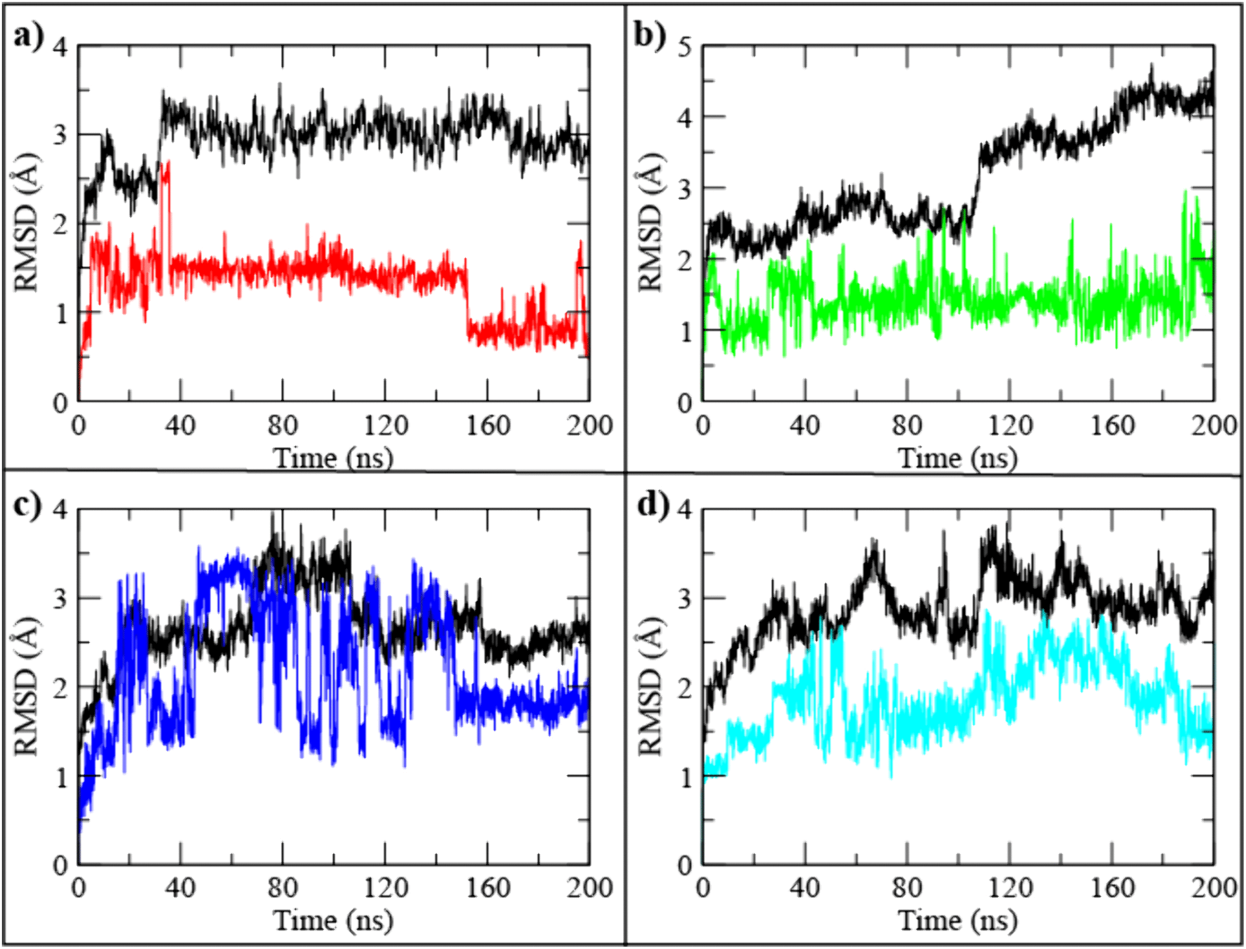
RMSD plots of SIRT4–ligand complexes over a 200 ns molecular dynamics simulation. (a) UBCS182, (b) CSC057320968, (c) PubChem-162316407, (d) ChemDiv-V013-1548. Protein RMSD is shown in black plot while ligands are shown by different colors.

Molecular dynamics (MD) simulations were conducted to evaluate the structural stability and conformational behavior of the top three SIRT4–ligand complexes CSC057320968, PubChem-162316407, and ChemDiv-V013-1548 in comparison with the reference inhibitor UBCS182 [34]. Each system was simulated for 200 ns under physiological conditions, and root mean square deviation (RMSD) analysis was used to monitor the temporal stability of the protein backbone and bound ligand throughout the trajectory. The UBCS182–SIRT4 complex (Fig 3a) displayed moderate backbone deviations, stabilizing around ~3.0–3.4 Å after ~40 ns. The ligand RMSD closely tracked the protein, indicating consistent retention in the binding pocket despite conformational drift. The CSC057320968–SIRT4 complex (Fig 3b) demonstrated the most stable profile, with backbone RMSD stabilizing between 2.2–2.8 Å throughout the trajectory. The ligand RMSD remained low (1.0–2.0 Å), indicating strong anchoring and limited positional displacement. For the PubChem-162316407 complex (Fig 3c), the backbone RMSD showed higher fluctuations (~2.5–3.5 Å) with noticeable drift after ~120 ns. Ligand RMSD exhibited intermittent oscillations, suggesting reorientation within the pocket but without dissociation. The ChemDiv-V013-1548 complex (Fig 3d) initially showed deviations, stabilizing between 2.8–3.2 Å after ~30 ns. Ligand RMSD remained below backbone RMSD for most of the trajectory, suggesting that the ligand adapted its orientation while maintaining persistent binding. Overall, the 200 ns RMSD analysis confirmed that all four complexes remained dynamically stable, but CSC057320968 exhibited the lowest fluctuations for both protein and ligand, making it the most conformationally stable candidate for further refinement and experimental validation.

#### 3.6.2 RMSF.

**Fig 4:**
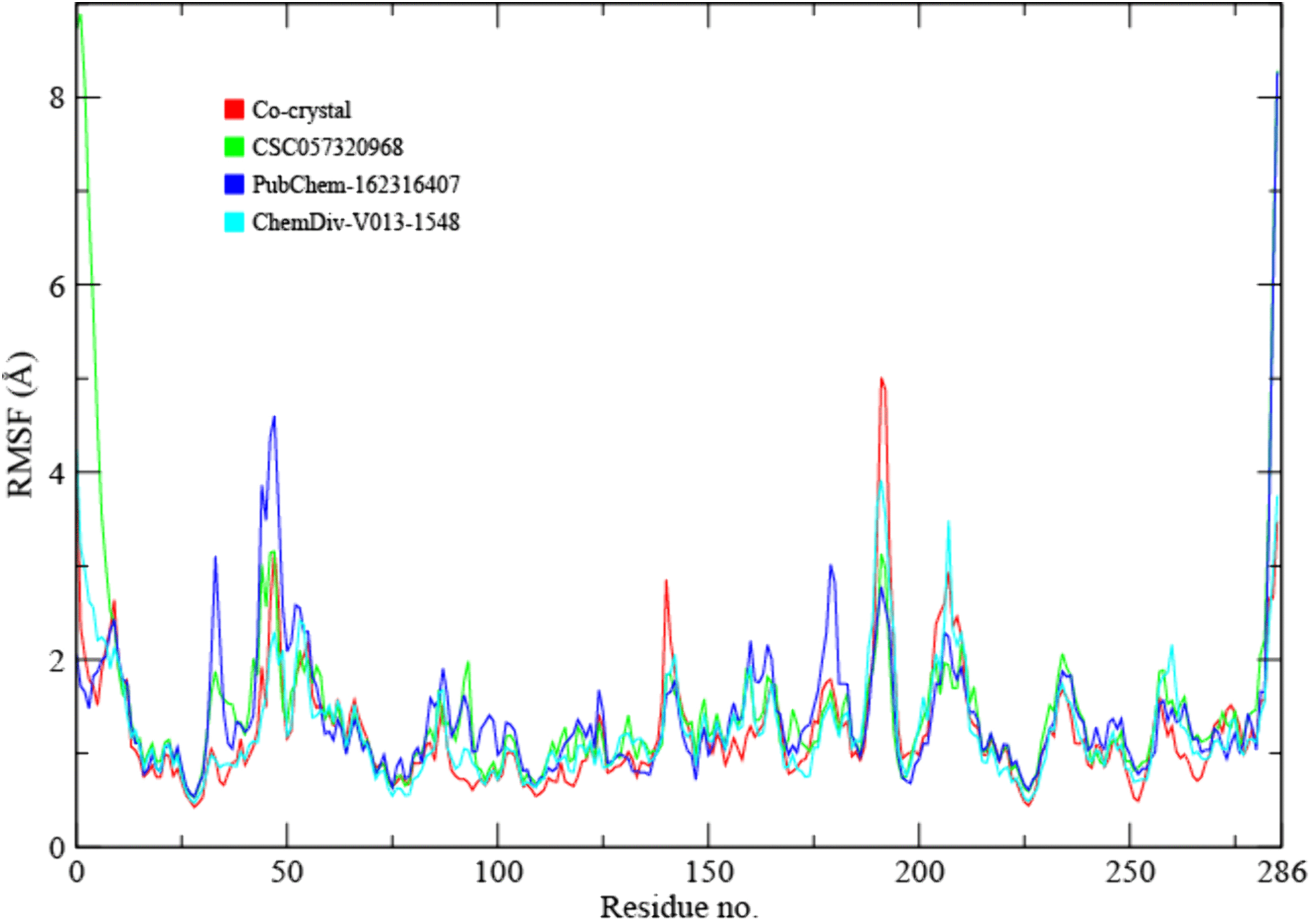
RMSF plots of SIRT4 residues across 200 ns simulations in the presence of ligands. Higher peaks indicate flexible loop or terminal regions, while low RMSF values represent stable, structured domains.

To evaluate the flexibility of SIRT4 residues during ligand binding, root mean square fluctuation (RMSF) analysis was performed on each residue over the 200 ns MD trajectory (Fig 4). RMSF measures the time-averaged atomic displacement of residues from their mean positions and is commonly used to identify flexible and rigid regions of proteins during simulations [35]. As expected, the reference UBCS182–SIRT4 complex showed higher fluctuations at the N-terminal (residues 1–40) and C-terminal (residues 250–275), which are solvent-exposed and less structured regions. Core secondary-structure elements around the binding site remained comparatively rigid (<2.0 Å). Binding of CSC057320968 resulted in the lowest overall fluctuations among the tested ligands. Notably, loop regions near residues 70–110 and 180–200 exhibited reduced flexibility compared to both UBCS182 and the other ligand complexes, suggesting that CSC057320968 contributes to stabilizing conformations surrounding the active site. The PubChem-162316407–SIRT4 complex showed higher fluctuations in the loop region spanning residues 60–80 and 170–200, with peaks exceeding 3.0 Å, indicative of increased local flexibility. The ChemDiv-V013-1548 complex also displayed moderate fluctuations, especially near residues 40, ~ 200, and ~260, consistent with terminal and loop mobility. Overall, RMSF profiles demonstrate that CSC057320968 induced the greatest conformational restraint across the protein backbone, further supporting its role as the most structurally stabilizing ligand among the tested candidates.

#### 3.6.3 Protein-ligand contacts.

**Fig 5:**
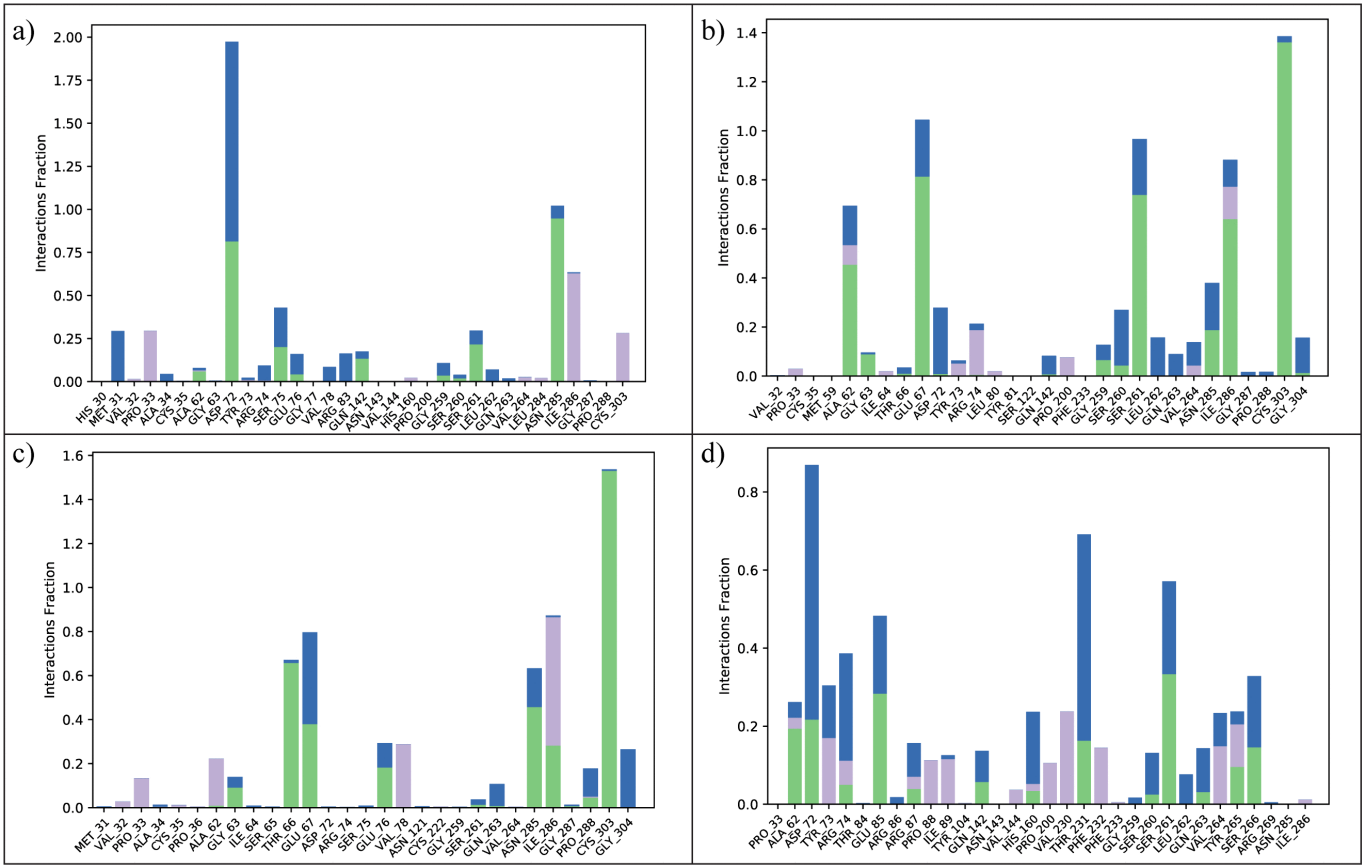
Residue interaction fingerprint plots during 200 ns MD simulations for four SIRT4–ligand complexes. (a) UBCS182, (b) CSC057320968, (c) PubChem-162316407, (d) ChemDiv-V013-1548. stacked bars indicate contributions from hydrogen bonds (green), hydrophobic contacts (blue), water bridges, and ionic interactions (purple). Higher bars denote more frequent and stable ligand-residue contacts.

To gain a comprehensive understanding of residue-level binding interactions throughout the simulation period, protein–ligand contact frequency analysis was conducted for all four complexes using the Simulation Interaction Diagram module. This analysis quantified the persistence and nature of interactions including hydrogen bonds, hydrophobic contacts, ionic bridges, and water-mediated bridges between the ligand and individual SIRT4 residues over the course of 200 ns. In the UBCS182–SIRT4 complex (Fig 5a), prominent and frequent interactions were observed with residues Arg74, Ser75, Asp72, Glu76, Gln142, Gly259, Ser261, and Asn285, all of which are located near or within the catalytic core of SIRT4. Notably, Arg74 exhibited the highest interaction fraction, primarily through persistent hydrogen bonds and electrostatic contacts. This suggests that UBCS182 relies heavily on charged and polar residues for tight binding. The CSC057320968–SIRT4 complex (Fig 5b) also demonstrated highly persistent interactions, particularly with Ala62, Gly63, Glu67, Ser260, Ser261, Asn285, Ile286, and Cys303. A distinct feature of this complex was the increased interaction frequency with backbone residues at the α-helix region flanking the active site, suggesting a more anchored binding orientation. Glu67 and Gly287 also showed high contact persistence, confirming that CSC057320968 maintains favorable hydrogen bonding and steric compatibility throughout the simulation. For the PubChem-162316407–SIRT4 complex (Fig 5c), critical contacts were observed with Gly63, Thr66, Glu67, Glu76, Asn285, Ile286, Pro288, and Cys303. Several of these interactions were mediated by hydrogen bonds, especially with backbone carbonyl and side-chain polar groups. While the overall contact distribution was slightly more scattered compared to CSC057320968, the frequent reoccurrence of contacts with residues like Pro33, Ser261, and Gly287 indicated consistent binding at the pocket interface. In the ChemDiv-V013-1548–SIRT4 complex (Fig 5d), residues Ala62, Asp72, Arg74, Glu85, His160, Thr231, Ser260, Ser261, Gln263, Tyr265, and Ser266 were identified as frequent contact contributors. This profile reflects a broader interaction footprint, possibly due to increased ligand flexibility or adaptation to pocket dynamics. Although individual contacts were moderately persistent, the overall breadth of interaction suggests a versatile ligand conformation. Altogether, these fingerprint profiles reinforce the MD stability results, where CSC057320968 demonstrated a more compact and residue-focused interaction network, correlating with its superior RMSD and RMSF behavior. UBCS182 showed the most intense individual residue interactions (e.g., Arg74), while ChemDiv-V013-1548 offered a wider but slightly less persistent contact spectrum.

#### 3.6.4 Radius of gyration (Rg) analysis.

**Fig 6:**
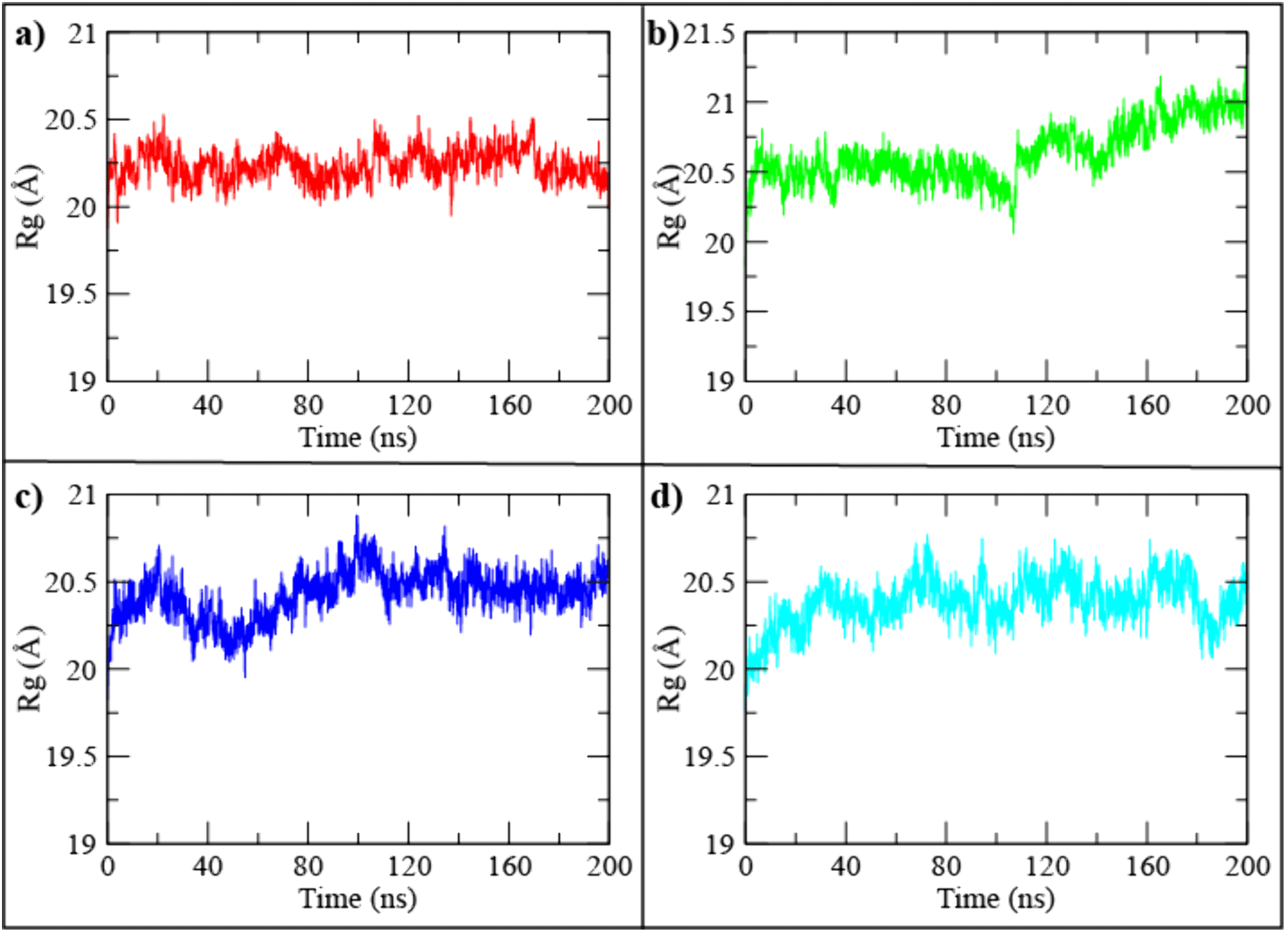
Radius of gyration (Rg) plots for the SIRT4–ligand complexes over 200 ns of molecular dynamics simulation. (a) UBCS182 (native inhibitor), (b) CSC057320968, (c) PubChem-162316407, and (d) ChemDiv-V013-1548.

To assess the overall structural compactness and folding behavior of SIRT4 in complex with each ligand, the radius of gyration (Rg) was monitored over the 200 ns trajectories (Fig 6a–d). Rg provides a measure of protein stability, with smaller fluctuations reflecting a more compact and stable conformation. The UBCS182–SIRT4 complex (Fig 6a) showed stable compactness throughout the simulation, with values fluctuating modestly between ~20.0 and 20.5 Å. The CSC057320968–SIRT4 complex (Fig 6b) displayed a similar trend, with Rg values consistently maintained in the 20.3–20.7 Å range, indicating minimal unfolding and strong structural retention. In contrast, the PubChem-162316407–SIRT4 complex (Fig 6c) exhibited a gradual upward drift in Rg beyond ~120 ns, reaching values above 20.8 Å, which may suggest increased flexibility or partial loosening of the protein structure. The ChemDiv-V013-1548–SIRT4 complex (Fig 6d) also showed an upward trend, though less pronounced than PubChem-162316407, suggesting moderate destabilization. Overall, the extended 200 ns simulations demonstrated that CSC057320968 maintained the most compact and stable protein fold, closely resembling the native inhibitor UBCS182, whereas PubChem-162316407 and ChemDiv-V013-1548 induced subtle increases in protein flexibility.

#### 3.6.5 Principal component analysis and free energy landscape.

**Fig 7:**
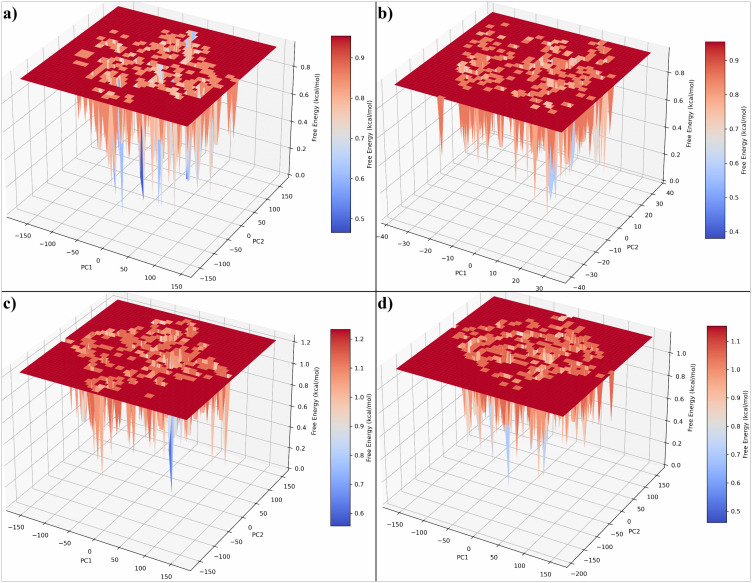
Three-dimensional free energy landscapes (FELs) projected along the first two principal components (PC1 and PC2) for the SIRT4–ligand complexes obtained from 200 ns molecular dynamics simulations. Panels represent (a) UBCS182 (native inhibitor), (b) CSC057320968, (c) PubChem-162316407, and (d) ChemDiv-V013-1548.

Principal component analysis (PCA) was performed to extract the dominant collective motions from the molecular dynamics trajectories of the SIRT4–ligand complexes. The first two principal components (PC1 and PC2) were used to construct the free energy landscapes (FELs), allowing visualization of the conformational space explored during simulation (Fig 7a–d). The FEL of the native complex SIRT4–UBCS182 (Fig 7a) displayed multiple shallow minima and a relatively broad energy basin, indicating that the system samples a wide range of conformations with moderate stability. The presence of several local minima suggests flexibility in the protein conformation upon binding to the native ligand. The complex with CSC057320968 (Fig 7b) revealed a more confined energy basin and a dominant low-energy well, suggesting a more restricted conformational landscape. This indicates that CSC057320968 stabilizes SIRT4 into fewer, energetically favorable conformations, implying strong binding affinity and conformational restraint. The FEL for PubChem-162316407 (Fig 7c) exhibited a more scattered energy distribution with higher energy fluctuations, reflecting significant structural rearrangements and reduced stability. Multiple shallow basins were observed, suggesting that this complex samples several semi-stable states without settling into a deep energy minimum. For ChemDiv-V013-1548 (Fig 7d), the FEL showed intermediate behavior between the native and the other analogs. It demonstrated a well-defined low-energy region surrounded by multiple local minima, implying moderate conformational flexibility and reasonably stable binding.

#### 3.6.6 State transition probability analysis.

**Fig 8:**
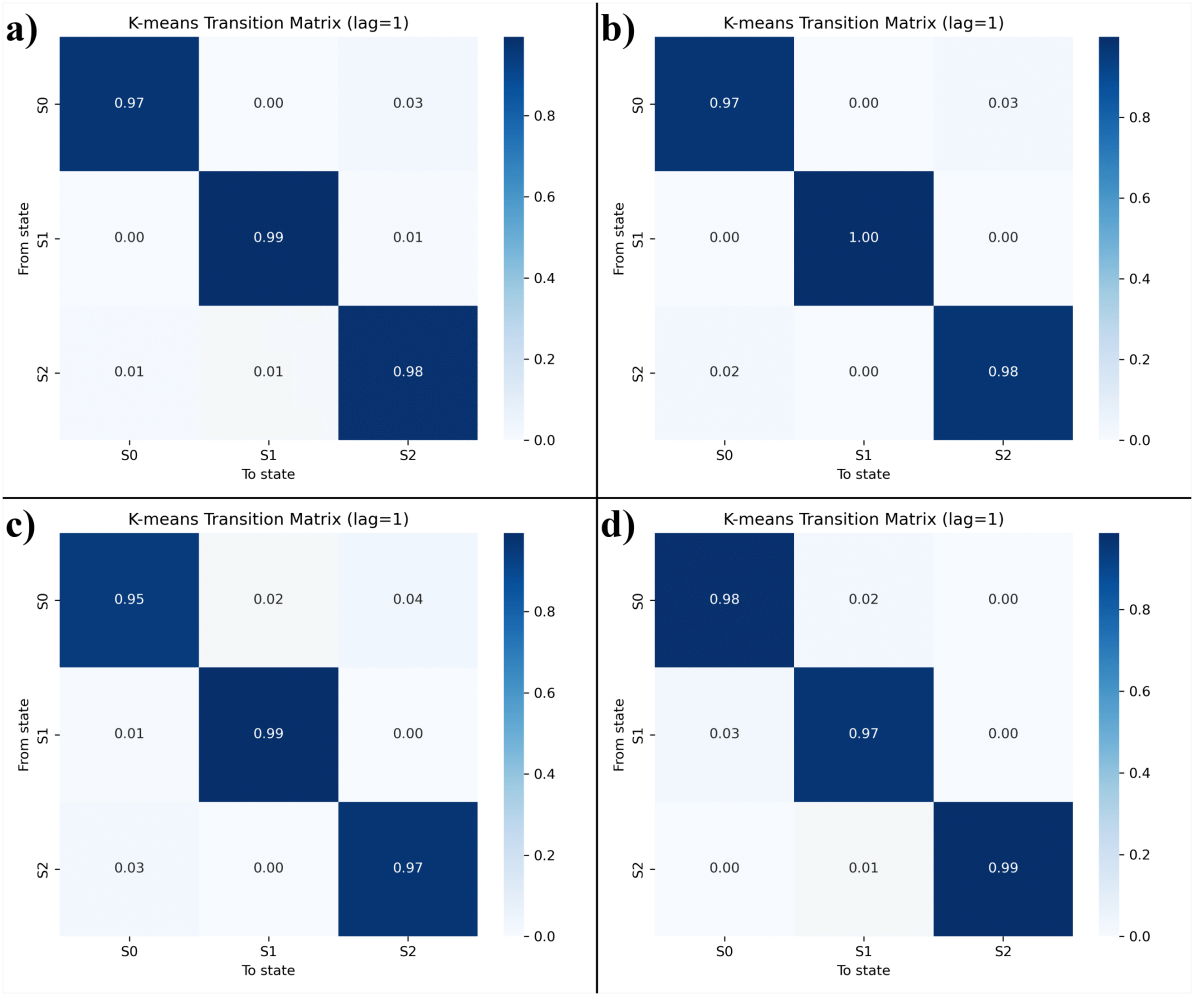
State transition probability matrices for SIRT4–ligand complexes derived from K-means clustering and Markov state modeling (MSM). (a) UBCS182–SIRT4, (b) CSC057320968–SIRT4, (c) PubChem-162316407–SIRT4, and (d) ChemDiv-V013-1548–SIRT4 complexes.

To further characterize conformational stability, Markov state modeling (MSM) combined with K-means clustering was performed to classify the SIRT4–ligand conformational space into three metastable states (S0–S2). The transition probability matrices at lag = 1 are shown in Fig 8a–d for each complex. The UBCS182–SIRT4 complex (Fig 8a) exhibited high self-transition probabilities (>0.97) across all states, with minimal cross-transitions (≤0.03). This indicates that the native complex remains stable in defined conformations, though it occasionally transitions between S0 and S2, consistent with the broad shallow minima observed in its FEL. The CSC057320968–SIRT4 complex (Fig 8b) demonstrated the strongest conformational retention. Self-transition probabilities of 0.97–1.00 were observed across all three states, with negligible inter-state transitions. This confirms that CSC057320968 restricts conformational sampling and stabilizes the protein into fewer energetically preferred conformations, in agreement with its confined FEL profile and lowest RMSD/Rg fluctuations. By contrast, the PubChem-162316407 complex (Fig 8c) showed slightly lower self-transition probabilities (0.95–0.99) and higher cross-transitions (~2–4%). This suggests greater conformational plasticity and supports the broader FEL distribution observed for this ligand. Similarly, the ChemDiv-V013-1548 complex (Fig 8d) showed strong state retention (≥0.97) but more frequent inter-state transitions (~2–3%) than CSC057320968, indicating moderate conformational flexibility. Overall, the state transition analyses demonstrate that CSC057320968 induces the most restricted and stable conformational ensemble, whereas PubChem-162316407 and ChemDiv-V013-1548 complexes sample more conformational states and undergo more frequent transitions. These results reinforce the conclusion that CSC057320968 is the most promising ligand for stable modulation of SIRT4 dynamics.

#### 3.6.7 MM/GBSA analysis.

To complement the MD simulations and evaluate binding energetics, binding free energies were calculated using the Prime MM/GBSA approach for the UBCS182 inhibitor and the top three ligands (CSC057320968, PubChem-162316407, and ChemDiv-V013-1548). The results are summarized in Table 7. The total binding free energies (ΔG_Bind_) were all strongly favorable, ranging from –87.67 to –103.30 kcal/mol, confirming stable ligand association with SIRT4. Among the tested ligands, ChemDiv-V013-1548 showed the most favorable binding energy (–103.30 kcal/mol), followed by PubChem-162316407 (–93.11 kcal/mol), UBCS182 (–90.91 kcal/mol), and CSC057320968 (–87.67 kcal/mol). Decomposition of the binding free energy terms revealed that van der Waals (ΔG_vdW_) and lipophilic contributions (ΔG_Lipo_) were the dominant stabilizing factors across all complexes. ChemDiv-V013-1548 demonstrated the strongest lipophilic (–52.76 kcal/mol) and van der Waals (–66.28 kcal/mol) interactions, consistent with its deep hydrophobic embedding in the binding pocket. Similarly, CSC057320968 displayed favorable van der Waals interactions (–67.84 kcal/mol) and balanced lipophilic contributions (–42.56 kcal/mol), supporting its stable binding profile observed in MD analyses. Electrostatic interactions (ΔG_Coulomb_) also contributed substantially, particularly in PubChem-162316407 (–29.01 kcal/mol) and ChemDiv-V013-1548 (–27.76 kcal/mol), while solvation penalties (ΔG_Solv-GB_) partially offset these stabilizing interactions, especially in ChemDiv-V013-1548 (42.50 kcal/mol). Hydrogen bonding and packing terms provided smaller but favorable contributions across all complexes, whereas covalent terms were marginally destabilizing. Overall, the MM/GBSA analysis highlights that ChemDiv-V013-1548 and CSC057320968 exhibit the most favorable binding energetics with SIRT4, primarily driven by van der Waals and hydrophobic interactions. These findings are consistent with the MD simulations and further support their prioritization as lead candidates for experimental validation.

**Table 7:** Prime MM/GBSA binding free energy decomposition for SIRT4–ligand complexes.

	UBCS182	CSC057320968	PubChem-162316407	ChemDiv-V013-1548
ΔG_Bind_	−90.90	−87.66	−93.11	−103.30
ΔG_Coulomb_	−18.80	−14.34	−29.01	−27.76
ΔG_Covalent_	5.630	8.610	9.285	3.467
ΔG_Hbond_	−1.03	−1.58	−3.59	−2.26
ΔG_Lipo_	−43.07	−42.55	−40.27	−52.76
ΔG_Packing_	−1.06	−0.19	−0.43	−0.214
ΔG_Solv-GB_	31.174	30.245	16.32	42.504
ΔG_vdW_	−63.73	−67.8	−45.40	−66.27

### 3.7 Quantum chemical descriptors

**Fig 9:**
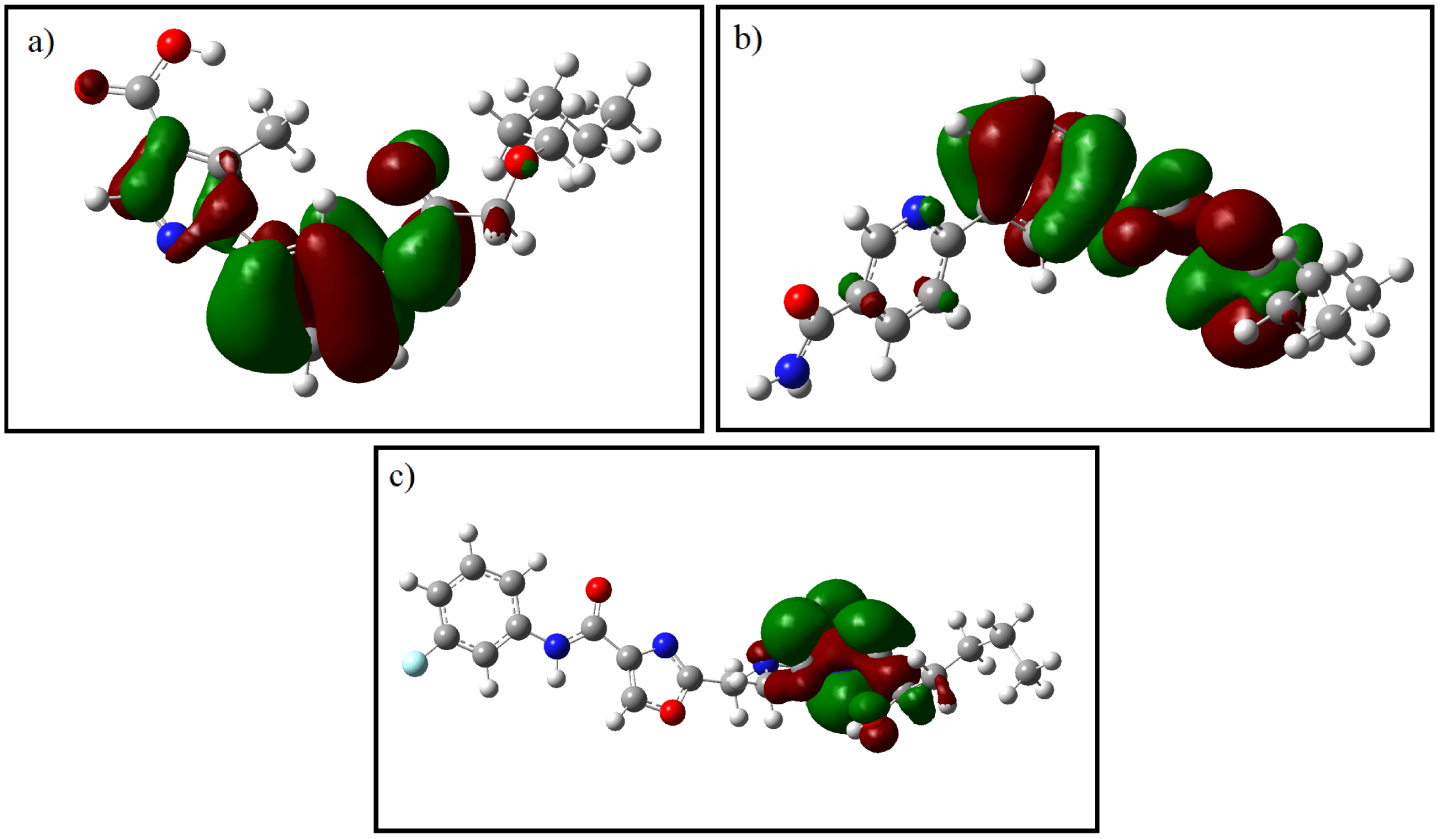
Frontier molecular orbital (FMO) distributions for the selected hit compounds: (a) CSC057320968, (b) PubChem-162316407, and (c) ChemDiv-V013-1548. Green and red lobes represent HOMO and LUMO surfaces, respectively.

Density Functional Theory (DFT) calculations were conducted to investigate the frontier molecular orbitals and global reactivity descriptors of the top three selected compounds (CSC057320968, PubChem-162316407, and ChemDiv-V013-1548). The Highest Occupied Molecular Orbital (HOMO) and Lowest Unoccupied Molecular Orbital (LUMO) distributions are shown in Fig 9, while calculated descriptors are summarized in Table 8. The analysis revealed that CSC057320968 exhibited a HOMO–LUMO gap of 5.41 eV, PubChem-162316407 had a gap of 4.22 eV, and ChemDiv-V013-1548 displayed a gap of 4.79 eV. These values suggest moderate electronic reactivity for CSC057320968 and PubChem-162316407, with a relatively lower gap than ChemDiv-V013-1548, which showed a higher HOMO–LUMO gap of 4.79 eV, suggesting greater chemical stability and lower reactivity. The calculated descriptors, including ionization potential, electron affinity, electronegativity, electrophilicity index, chemical hardness, and chemical softness, further highlight the differences in the electronic properties of the compounds. CSC057320968 demonstrated the highest chemical hardness (5.86 eV), indicating relatively stable electronic behavior compared to PubChem-162316407 and ChemDiv-V013-1548. Both CSC057320968 and PubChem-162316407 exhibited favorable ADMET properties, including good solubility, acceptable LogP values, and low mutagenicity flags, in line with Lipinski’s Rule of Five (LRO5), suggesting they retain good bioavailability and drug-likeness despite their increased reactivity. The reactivity profile of these compounds suggests that while they exhibit higher electronic reactivity compared to ChemDiv-V013-1548, their ADMET properties offset concerns regarding potential toxicity. In particular, CSC057320968, with a moderate electronegativity value (6.79 eV) and chemical softness (0.17 eV), presents a favorable electronic profile and is expected to interact adaptively within the active site of SIRT4, making it a suitable candidate for further optimization. These findings emphasize the importance of considering quantum chemical descriptors alongside pharmacokinetic properties to prioritize lead compounds for further development.

**Table 8:** Frontier molecular orbital energies and global reactivity descriptors calculated by using DFT analysis.

	CSC057320968	PubChem-162316407	ChemDiv-V013-1548
E_HOMO_	−6.33 eV	−5.56 eV	−5.78 eV
E_LUMO_	−0.92 eV	−1.34 eV	−0.99 eV
Ionization Potential	6.33 eV	5.56 eV	5.78 eV
Electron Affinity	0.92 eV	1.34 eV	0.99 eV
Electronegativity	6.79 eV	6.24 eV	6.27 eV
Electrophilicity Index	−6.79 eV	−6.24 eV	−6.27 eV
Chemical Hardness	5.86 eV	4.89 eV	5.28 eV
Chemical Softness	0.17 eV	0.20 eV	0.18 eV

## 4. Discussion

Diabetic nephropathy (DN) continues to pose a major clinical challenge as a leading cause of end-stage renal disease, yet therapeutic strategies remain limited due to the complex interplay of oxidative stress, inflammation, and mitochondrial dysfunction [36,37]. Among the molecular regulators of mitochondrial homeostasis, Sirtuin 4 (SIRT4) has gained interest for its role in mitigating ROS, apoptosis, and metabolic dysregulation in renal tissues [7,8,38]. Despite its biological significance, no selective or clinically validated small-molecule modulators of SIRT4 currently exist. This research addressed that critical gap through a structure-based virtual screening pipeline integrating pharmacophore modeling, molecular docking, ADMET profiling, molecular dynamics (MD) simulations, and density functional theory (DFT) analysis to identify novel SIRT4 inhibitors with therapeutic potential in DN.

The central hypothesis driving this study was that a pharmacophore-based approach, combined with modern computational modeling, could enable the rational discovery of drug-like ligands targeting SIRT4. The most significant finding of this study is the identification of three structurally diverse small molecules CSC057320968, PubChem-162316407, and ChemDiv-V013-1548 that exhibited high binding affinity to SIRT4, along with favorable ADMET properties and stable binding behavior in MD simulations. This pipeline validated CSC057320968 as the most promising compound based on RMSD, RMSF, Rg, residue contact persistence, and DFT-based descriptors such as chemical softness and HOMO energy levels. These findings represent a novel contribution, as very few studies to date have combined these orthogonal computational layers to identify SIRT4 modulators [9,14,15].

Pharmacophore modeling based on the known inhibitor UBCS182 captured the core interaction features required for SIRT4 binding, and virtual screening of 3,285 hits yielded top-ranked candidates with docking scores between −9.46 and −8.41 kcal/mol. Previous efforts to identify sirtuin modulators have mostly targeted SIRT1, SIRT3, or SIRT6 [39–41], with limited structural or functional exploration of SIRT4 in drug discovery efforts. By filling this gap, our study broadens the sirtuin inhibitor landscape and introduces novel scaffolds that are chemically and pharmacokinetically viable. The interaction fingerprints revealed sustained hydrogen bonding and hydrophobic contacts with catalytic residues such as Arg74, Ser75, and Cys303—residues previously implicated in enzymatic function and stability [10,42].

Notably, DFT analysis provided an additional layer of validation, revealing differences in electronic reactivity profiles among the three hit compounds. For example, CSC057320968 exhibited a moderate electron-donating capacity (E_HOMO_: −6.33 eV) and chemical softness (0.17 eV), suggesting adaptability to the polar environment of the active site. In contrast, PubChem-162316407 and ChemDiv-V013-1548 displayed slightly lower electron-donating capabilities (E_HOMO_: −5.56 eV and −5.78 eV, respectively) but also demonstrated favorable reactivity profiles. These findings suggest that CSC057320968, with its higher chemical softness and moderate HOMO value, might be more reactive, making it adaptable for interactions with the SIRT4 active site while maintaining an acceptable safety margin. Similar applications of quantum chemical descriptors have previously shown predictive power in ligand–target compatibility and biological activity prediction [43,44]. These findings further support the inclusion of DFT metrics in virtual screening pipelines aimed at redox-sensitive or mitochondrial proteins like SIRT4.

Secondary results from the 200 ns MD simulations strengthened the case for CSC057320968. Compared to UBCS182 and other candidates, CSC057320968 induced lower conformational fluctuations (RMSF < 2 Å), more consistent compactness (Rg ~ 20.3 Å), and tighter residue-level contacts, indicating better dynamic stabilization. This aligns with past literature showing that MD-derived stability metrics strongly correlate with in vitro activity and bioavailability [45,46]. The free energy landscape (FEL) of the CSC057320968–SIRT4 complex further suggested a narrow and energetically favorable conformational space, a hallmark of specific and high-affinity ligand binding [47].

However, our study is not without limitations. First, while the computational pipeline is rigorous, all results remain predictive in nature, and biological validation (e.g., enzyme inhibition assays, cellular models) is required to confirm SIRT4 binding and functional modulation. Second, only one known SIRT4 inhibitor (UBCS182) was available to train the pharmacophore, potentially limiting chemical diversity during screening. Third, while ADMET predictions identified candidates with low mutagenicity and good drug-likeness, these models cannot fully capture in vivo metabolic complexity or long-term toxicity [48–50].

Future research should focus on experimental validation of CSC057320968 and analogs in enzymatic and cell-based assays to evaluate their efficacy in mitigating podocyte injury or mitochondrial stress. Structure–activity relationship (SAR) studies based on the identified scaffolds could optimize potency, and cryo-EM or X-ray crystallography may confirm binding modes. Additionally, systems pharmacology modeling could elucidate off-target effects and systemic impact in DN models. Given the mitochondrial specificity of SIRT4, downstream assays should also include ROS profiling, mitochondrial membrane potential assays, and expression analysis of pro-inflammatory cytokines and apoptosis markers.

## 5. Conclusion

In conclusion, this study presents a first-of-its-kind computational pipeline targeting SIRT4 for diabetic nephropathy. By combining pharmacophore-guided screening, molecular dynamics, and quantum chemistry, we identified structurally novel, stable, and pharmacokinetically viable SIRT4 inhibitors. Our work addresses the current void in mitochondrial drug discovery for DN and offers a replicable framework for future targeting of sirtuins and other redox-sensitive enzymes in metabolic diseases. Looking ahead, the most promising candidate, CSC057320968, should be prioritized for experimental validation. Initial assays could include in vitro enzymatic activity measurements to confirm inhibition of recombinant human SIRT4, followed by in vitro podocyte or proximal tubular cell models of diabetic nephropathy to assess its effects on mitochondrial function, ROS generation, and apoptosis. Further evaluation in ex vivo kidney tissue assays or in vivo DN rodent models would establish therapeutic potential and pharmacokinetic behavior. Such follow-up studies would provide crucial biological confirmation of the computational predictions and advance CSC057320968 toward preclinical development.
